# Selection of parameters for thermal coronavirus inactivation – a data-based recommendation

**DOI:** 10.3205/dgkh000351

**Published:** 2020-07-13

**Authors:** Martin Hessling, Katharina Hoenes, Christian Lingenfelder

**Affiliations:** 1 Institute of Medical Engineering and Mechatronics, Ulm University of Applied Sciences, Ulm, Germany; 2 Pharmpur GmbH, Koenigsbrunn, Germany

**Keywords:** coronavirus, SARS-CoV, SARS-CoV-2, MERS-CoV, heat, thermal inactivation, sterilization

## Abstract

**Background:** Healthcare workers and large parts of the population are currently using personal protective equipment, such as face masks, to avoid infections with the novel coronavirus SARS-CoV-2. This equipment must be sterilized as gently as possible before reuse. One possibility is thermal inactivation, but professional autoclaves with their high temperatures are often not available or suitable. If the inactivation period is long enough, coronavirus inactivation can also be carried out at relatively low temperatures. The required duration was determined in this study.

**Material and methods:** Data from published thermal inactivation studies on coronaviruses were applied to determine the temperature dependence of the rate constant k(T) for each coronavirus by employing Arrhenius models.

**Results:** The data obtained exhibit large variations, which appear to be at least partially caused by different sample properties. Samples with high protein content or samples in dry air sometimes seem to be more difficult to inactivate. Apart from this, the Arrhenius models describe the thermal inactivation properties well and SARS-CoV and SARS-CoV-2 can even be represented by a combined model. Furthermore, the available data suggest that all samples, including critical ones, can be mathematically included by a worst-case Arrhenius model.

**Conclusion:** Coronaviruses can already be inactivated at relatively low temperatures. For most samples, application times of approximately 32.5, 3.7, and 0.5 minutes will be sufficient at 60°C, 80°C, and 100°C, respectively, for a 5 log-reduction. For difficult conditions, the worst-case model provides significantly longer application times of 490, 55, and 8 minutes for the temperatures mentioned.

## Zusammenfassung

**Hintergrund:** Um Infektionen mit dem neuen Coronavirus SARS-CoV-2 zu vermeiden, setzen sowohl Beschäftigte im Gesundheitswesen als auch große Teile der Bevölkerung derzeit persönliche Schutzausrüstung, wie Gesichtsmasken, ein. Diese Ausrüstung muss vor der Wiederverwendung möglichst schonend sterilisiert werden. Eine Möglichkeit dafür ist die thermische Inaktivierung. Professionelle Autoklaven mit ihren hohen Temperaturen sind jedoch oft nicht verfügbar oder geeignet. Bei Coronaviren kann die Inaktivierung aber auch bei niedrigeren Temperaturen erfolgen, wenn die Inaktvierungsdauer lang genug ist. Diese notwendige Dauer soll hier bestimmt werden.

**Material und Methoden:** Daten aus publizierten thermischen Inaktivierungsstudien an Coronaviren werden herangezogen, um mit Hilfe von Arrhenius-Modellen für jedes Coronavirus die Temperaturabhängigkeit der Ratenkonstante k(T) zu bestimmen.

**Ergebnisse:** Die ermittelten Daten weisen große Variationen auf, die möglicherweise auch auf unterschiedliche Probeneigenschaften zurückzuführen sind. Coronaviren in Proben mit hohem Proteingehalt oder Proben an trockener Luft scheinen manchmal schwerer zu inaktivieren sein. Abgesehen davon scheint das Arrhenius-Modell die thermischen Inaktivierungseigenschaften gut zu beschreiben, wobei sich SARS-CoV und SARS-CoV-2 durch ein gemeinsames Modell darstellen lassen. Darüber hinaus deuten die verfügbaren Daten darauf hin, dass alle Proben mathematisch durch ein Worst-Case-Modell berücksichtigt werden können, das auch potentiell kritische Proben miteinschließt.

**Schlussfolgerung:** Coronaviren lassen sich bereits durch relativ niedrige Temperaturen inaktivieren. Für Standardproben sind z.B. bei 60°C, 80°C und 100°C Anwendungsdauern von ca. 32,5, 3,7 und 0,5 min notwendig, um eine Reduktion von 5 Logstufen zu erreichen. Für eine sicherere Inaktivierung liefert das Worst-Case-Modell für die genannten Temperaturen deutlich längere Anwendungszeiten von 490, 55 und 8 min.

## Introduction

The emergence of the novel coronavirus SARS-CoV 2 in Wuhan (China) in 2019 has led to a pandemic that is still spreading. In order to contain its further expansion, personal protective equipment, such as face masks, is increasingly being used both in the healthcare system and among the population. This sudden rise in demand has led to a worldwide shortage, so that even disposable items are sometimes used several times [1], [2], [3]. This leads to the question of how coronaviruses in or on such disposables, but also in or on home-made or professionally manufactured fabric masks or other solid or liquid materials, including waste and human samples, can be inactivated as gently and effectively as possible?

Thermal inactivation, which in the professional sector is often carried out with steam sterilizers at temperatures of up to 121°C or even 135°C, has long been known as a very effective disinfection technique for a wide range of pathogens [4], [5], [6]. However, this approach with its typically high temperatures is not suitable for all materials and is not available for many users.

A recent overview on coronavirus inactivation temperatures by Kampf et al. [7], as well as the first corresponding investigations on SARS-CoV-2 [8], [9], [10], [11], [12], [13], [14], reveal that coronaviruses can also be inactivated by temperatures well below 120°C. Even temperatures of 60°C or lower can reduce the coronavirus load, in which the duration of the heat application increases with decreasing temperature.

In a simple first-order reaction model, the relationship between virus concentration c(t) and time t can be described by an exponential function with the rate constant k [15], [16]. 

**Equation 1**

**
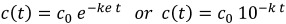
** **
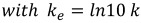
**

**Figure 1:**
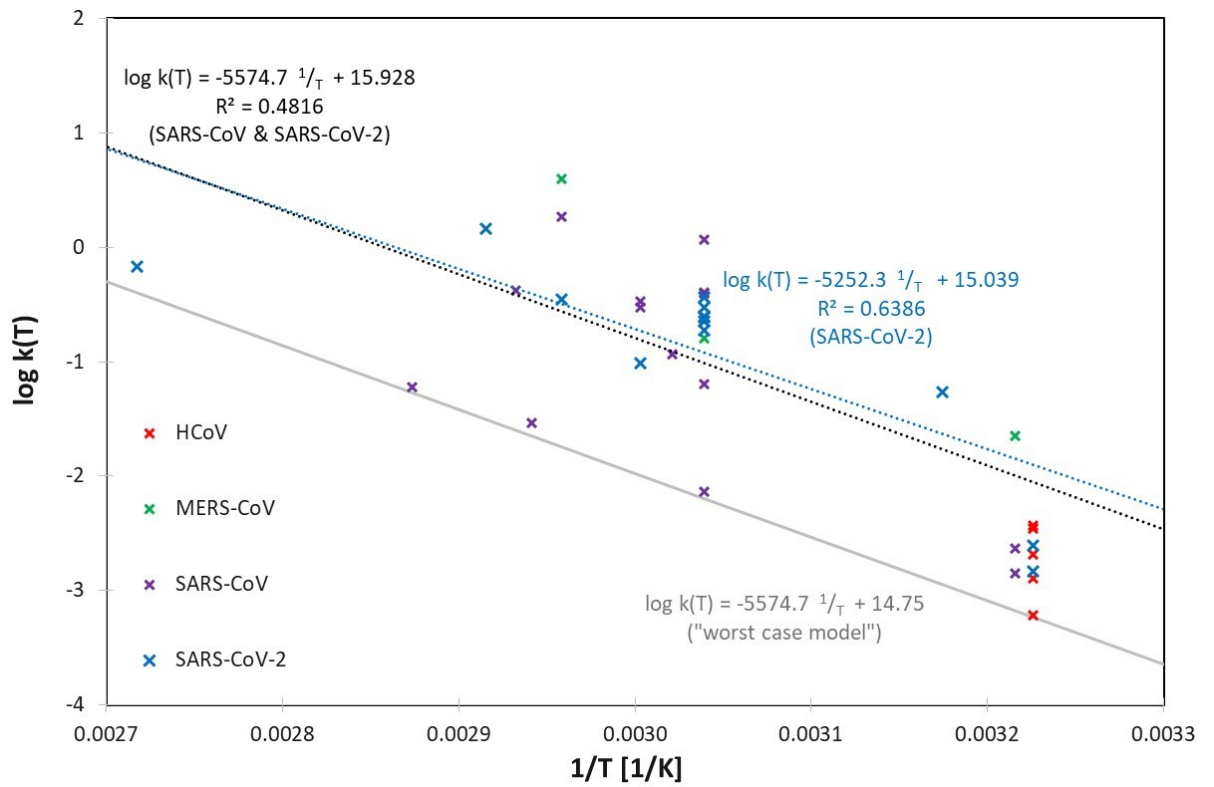
Logarithmic representation of the determined rate constants log k(T) for the human coronaviruses SARS-CoV, SARS-CoV-2, MERS-CoV and HCoV, together with linear regressions for SARS-CoV-2 and SARS-CoV & SARS-Co-V-2 together. A “worst-case model” was generated by shifting the SARS-CoV & SARS-CoV-2 regression curve, so that all determined rate constants for human coronaviruses are just above this line.

**Figure 2:**
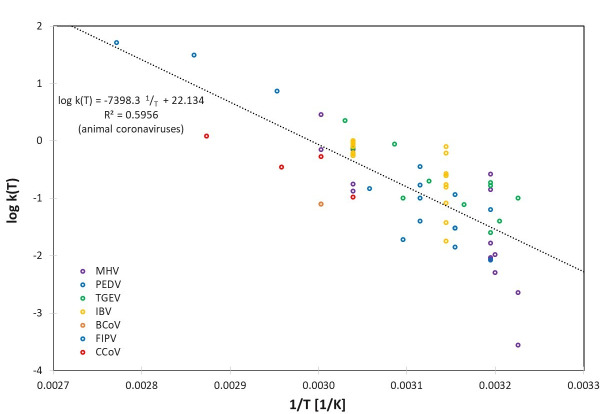
Logarithmic representation of the determined rate constants log k(T) for the animal coronaviruses MHV, PEDV, TGEV, IBV, BCoV, FIPV and CCoV together with a combined linear regression for log k(T) for all animal coronaviruses

For coronaviruses, this can be observed particularly well in the experimental data of Hofmann and Wyler ([17] Fig. 2), Daniel and Talbot ([18], Fig. 2) or Laude ([19], Fig. 1), and this relationship was also applied in the coronavirus investigations of Laude [19] and Liu [20].

The rate constant k=k(T) is a function of the inactivation temperature T, which can be mathematically described by an Arrhenius approach [15], [16]. This has also successfully been applied in previous coronavirus studies [19], [20]. In short, this approach describes k(T) as an exponential function of the temperature and two virus-dependent parameters, a and b.

**Equation 2**


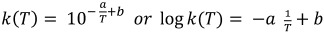


In this expression, log k(T) is represented by a straight line with the slope –a, the variable 1/T and the intercept b. The aim of this paper was to apply this mathematical model for calculating rate constants based on published coronavirus inactivation data, and use them to recommend temperature-dependent inactivation durations.

## Material and methods

PubMed and Google Scholar were used to search for different combinations of the terms coronavirus, heat, temperature, inactivation, disinfection and sterilization. The references of the publications found in this process were checked for further relevant studies. Since the focus of this investigation was on heat inactivation, only data with temperatures above about 40°C and pH values between 6 and 8 were included in the further analysis, since higher and lower pH values already lead to coronavirus inactivation without additional heating [17], [18], [21], [22].

If the authors did not provide rate constants themselves, the log-reduction and the required exposure time were determined from text, tables or figures to calculate the rate constant k. If data were available for several exposure durations, as a rule, the longest exposure at which a coronavirus concentration above the detection limit was still found was chosen. The reason for this is that the heating and cooling of the investigated samples – before and after reaching the intended temperature – also contribute to inactivation, but this is not recorded separately and their relative proportions are higher for short exposures than for longer ones.

The rate constants thus obtained for different temperatures were then applied to generate equations for k(T) for the different coronaviruses according to the Arrhenius model.

## Results and discussion

About 35 publications on thermal inactivation of the human viruses human coronavirus (HCoV), Middle East respiratory syndrome coronavirus (MERS-CoV), severe acute respiratory syndrome coronavirus (SARS-CoV) and severe acute respiratory syndrome coronavirus 2 (SARS-CoV-2) were found. Additionally, inactivation data for the following animal viruses was available: bovine coronavirus (BCoV), canine coronavirus (CCoV), feline infectious peritonitis coronavirus (FIPV), infectious bronchitis coronavirus (IBV), murine coronavirus (MHV), porcine epidemic diarrhea virus (PEDV), and transmissible gastroenteritis virus (TGEV). The studies contain approx. 120 data sets that met the above-mentioned selection criteria with regard to temperature and pH. However, not all of them could be evaluated quantitatively, as in some cases, only lower limits for the achieved log-reduction are given. 

Attachment 1 provides an overview of the obtained data, sorted by coronavirus and inactivation temperature.

The data exhibit large variability. The rate constants can differ by an order of magnitude even for one temperature and one coronavirus species. Some of these variations are probably caused by experimental inaccuracies. This can be assumed, for example, from the fact that in some experiments the determined virus concentration not only decreased but also increased again at certain phases during incubation. A further experimental factor is the effect of the heating and cooling phases already mentioned above, which can be particularly noticeable for short inactivation periods. Strain differences also contribute to this variability [19], [23], [24].

Another aspect tat has been observed in earlier publications is the influence of medium or environment. In liquid media, higher protein concentrations often seem to reduce virus inactivation [25], [26], [27] but exceptions also appear to exist [28]. Similar influences of environmental factors are observed in experiments on surfaces, which differ when performed at varying humidities, with low humidities impeding inactivation [29], [30], [31].

These large variations can also be observed in the logarithmic representation of the determined rate constants log(k), which are plotted separately for human and animal coronaviruses in Figure 1 (Fig. 1) and Figure 2 (Fig. 2) using the reciprocal of temperature 1/T (in Kelvin).

Apart from the variabilities mentioned above, and the differences for the individual coronavirus species represented in Table 1 (Tab. 1), the rate constants k(T) seem to be described relatively well by the assumed Arrhenius model. In Figure 2 (Fig. 2), even a single set of parameters appears to be suitable to describe all 7 different animal coronavirus species.

Figure 1 (Fig. 1) illustrates the behavior of the three human coronaviruses for which data on different inactivation temperatures are available. As in the analysis of animal coronaviruses, it is also consistent with the assumption of an Arrhenius model. MERS-CoV seems to be slightly more temperature sensitive, but the data of SARS-CoV and SARS-CoV-2, which belong to the same betacoronavirus subgenus sarbecovirus, exhibit similar temperature behaviors and can even be well described by a combined Arrhenius model. If the straight line belonging to this model is shifted downwards, which corresponds to an assumed reduction of the rate constant, a mathematical description for k(T) is obtained that describes a worst-case scenario, e.g., with inactivation under particularly unfavorable conditions.

With the data for k(T) from Table 1 (Tab. 1) the inactivation duration for a selected temperature T and a desired log-reduction LR can be calculated by

**Equation 3**


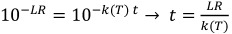


As an example of the application of these models, Table 2 (Tab. 2) provides necessary inactivation durations for a targeted 5 log-reduction for SARS-CoV or SARS-CoV-2 at different temperatures under assumed standard and worst-case conditions. (The temperature T must be entered in degrees Kelvin, which is the temperature in degrees Celsius plus 273.) 

## Conclusions

All investigations prove that coronaviruses can be inactivated quite quickly at relatively low temperatures, e.g., 60°C or 80°C. These are temperatures for which no professional autoclaves are necessary, but which can even be achieved with domestic cooking equipment such as an oven or rice cooker.

The observed temperature dependence of coronavirus inactivation behavior – represented by the rate constant k(T) – is well described by Arrhenius models, so that necessary inactivation times can be calculated for each coronavirus and each inactivation temperature. 

The two particularly important and related human coronaviruses SARS-CoV and SARS-CoV-2 behave very similarly and can be described by a combined Arrhenius model. This model delivers inactivation durations of approx. 32.5, 3.7 and 0.5 minutes for temperatures of 60°C, 80°C and 100°C, respectively, for a 5 log-reduction. For potentially critical samples the proposed worst-case model should be applied for safety reasons. 

## Notes

### Competing interests

The authors declare that they have no competing interests.

## Supplementary Material

Attachment 1: Coronavirus inactivation data by coronavirus and temperature

## Figures and Tables

**Table 1:**
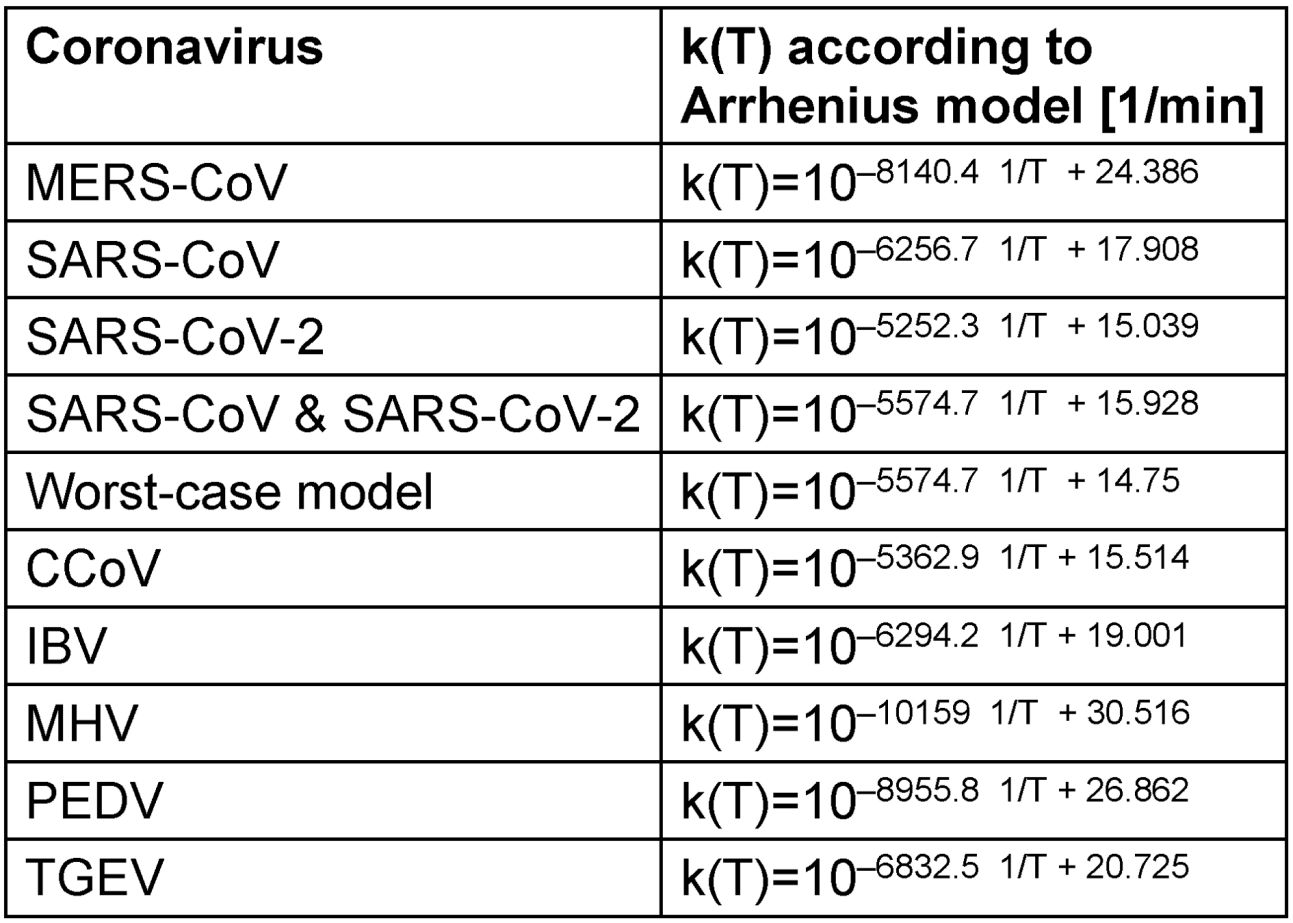
Mathematical description of the rate constants k(T) for the different coronaviruses based on the Arrhenius model and linear regression for log k(T)

**Table 2:**
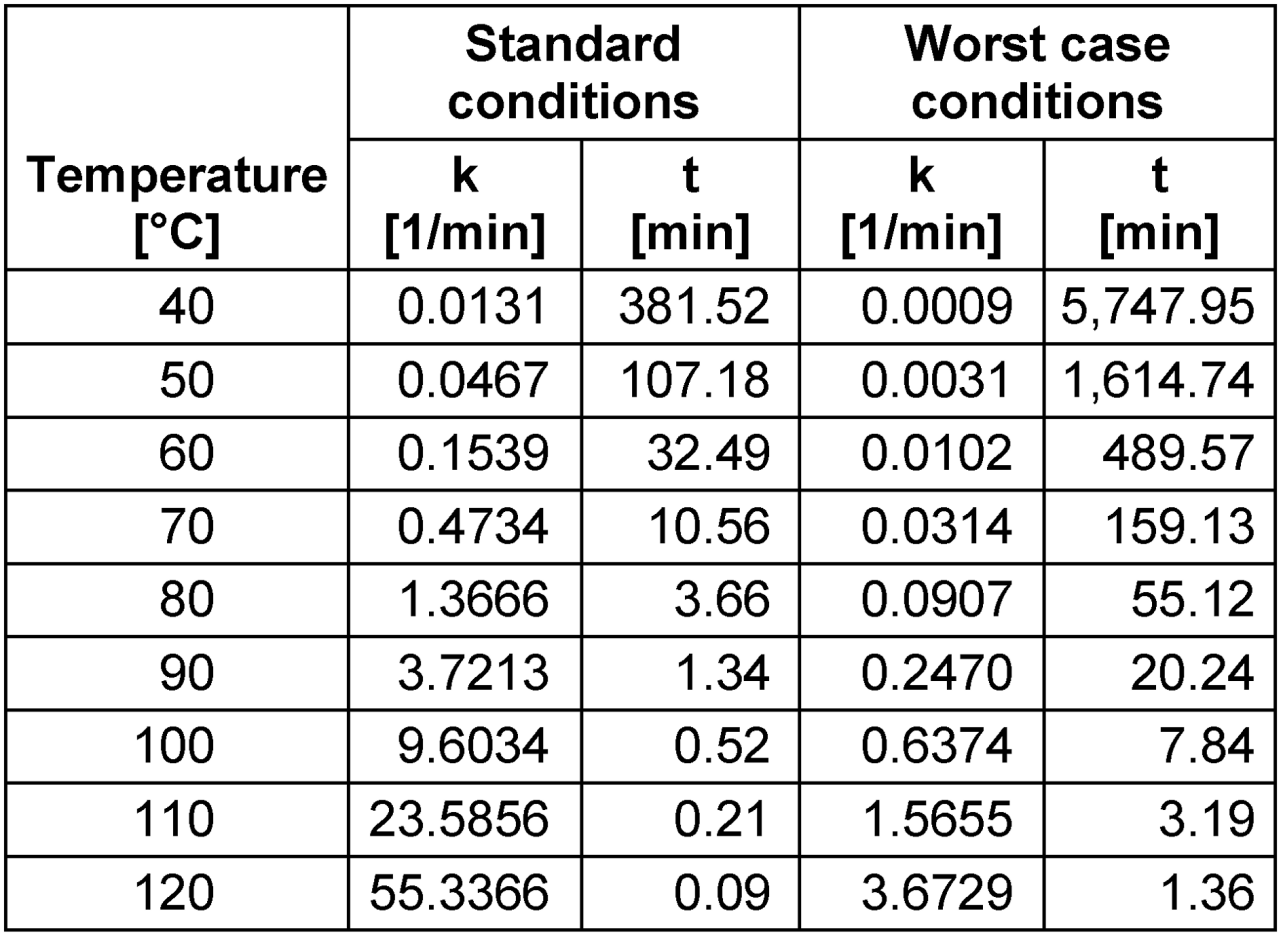
Necessary inactivation durations for thermal SARS-CoV or SARS CoV-2 inactivation for standard and worst-case conditions
