# Supplementary material for: Selection of parameters for thermal coronavirus inactivation – a data-based recommendation
Source: GMS Hyg Infect Control. 2020 Jul 13;15:Doc16. doi: 10.3205/dgkh000351 (PMC7373095; doi:10.3205/dgkh000351)
Supplement: Attachment 1: Coronavirus inactivation data by coronavirus and temperature [file HIC-15-16-s-001.pdf]

Attachment 1: Coronavirus inactivation data by coronavirus and temperature

Virus	Temperature [°C]	Exposition duration [min]	Log-reduction	k [1/min]	Sample condition	Remark	Reference*
Bovine coronavirus (BCoV American)	60	60	4.7	0.0783	liquid (EMEM +10% FCS)	values taken from Tab. III	[32]
Bovine coronavirus (BCoV Kakegawa)	60	60	4.7	0.0783	liquid (EMEM +10% FCS)	values taken from Tab. III	[32]
Bovine coronavirus (BCoV G110)	63	5	>6		liquid (sterilized milk)	values taken from text, not included in analysis	[33]
Bovine coronavirus (BCoV G110)	72	0.25	>6		liquid (sterilized milk)	values taken from text, not included in analysis	[33]
Canine coronavirus (CCoV S378)	56	50	5.25	0.1050	liquid (EMEM +10% FCS)	values taken from Fig. 1	[34]
Canine coronavirus (CCoV I-71)	60	5	2.64	0.5280	liquid (MEM +2% FCS)	values taken from Tab. 3	[35]
Canine coronavirus (CCoV S378)	65	15	5.26	0.3507	liquid (EMEM +10% FCS)	values taken from Fig. 1	[34]
Canine coronavirus (CCoV S378)	75	5	6.07	1.2140	liquid (EMEM +10% FCS)	values taken from Fig. 1	[34]
Feline infectious peritonitis coronavirus (FIPV WT/DF2)	54	20	2.97	0.1485	liquid (BME +5% FCS)	values taken from Fig. 2	[36]
Human coronavirus (HCoV-229E)	37	5,760	3.48	0.0006	liquid (EMEM +5% FCS)	values taken from Fig. 1 (day 1-5)	[21]
Human coronavirus (HCoV-229E)	37	600	2.23	0.0037	liquid (EMEM +2% FCS)	values taken from Fig. 1	[37]
Human coronavirus (HCoV-229E)	37	660	2.29	0.0035	liquid (EMEM +0,2% BSA)	values taken from Fig. 5	[37]
Human coronavirus (HCoV-OC43)	37	1,440	1.84	0.0013	liquid (EMEM +2% FCS)	values taken from Fig. 1	[37]
Human coronavirus (HCoV-OC43)	37	1,500	3.07	0.0020	liquid (EMEM +0,2% BSA)	values taken from Fig. 5	[37]
Infectious bronchitis coronavirus (IBV A-5968)	45	15	2.57	0.1713	liquid (diluted medium)	values taken from Fig. 2	[23]
Infectious bronchitis coronavirus (IBV Be-42)	45	5	4	0.8000	liquid (diluted medium)	values taken from Fig. 2	[23]

Attachment to: Hessling M, Hoenes K, Lingenfelder C. Selection of parameters for thermal coronavirus inactivation – a data-based recommendation. GMS Hyg Infect Control. 2020;15:Doc16. DOI: 10.3205/dgkh000351
*References refer to the article

Virus	Temperature [°C]	Exposition duration [min]	Log-reduction	k [1/min]	Sample condition	Remark	Reference*
Infectious bronchitis coronavirus (IBV Connaught)	45	15	2.33	0.1553	liquid (diluted medium)	values taken from Fig. 2	[23]
Infectious bronchitis coronavirus (IBV Holte)	45	15	4	0.2667	liquid (diluted medium)	values taken from Fig. 2	[23]
Infectious bronchitis coronavirus (IBV IB-41)	45	15	0.57	0.0380	liquid (diluted medium)	values taken from Fig. 2	[23]
Infectious bronchitis coronavirus (IBV Iowa-609)	45	15	3.71	0.2473	liquid (diluted medium)	values taken from Fig. 2	[23]
Infectious bronchitis coronavirus (IBV Ishida)	45	15	3.71	0.2473	liquid (diluted medium)	values taken from Fig. 2	[23]
Infectious bronchitis coronavirus (IBV KH)	45	15	0.27	0.0180	liquid (diluted medium)	values taken from Fig. 2	[23]
Infectious bronchitis coronavirus (IBV Nerima)	45	5	3.05	0.6100	liquid (diluted medium)	values taken from Fig. 2	[23]
Infectious bronchitis coronavirus (IBV Shiga)	45	15	1.24	0.0827	liquid (diluted medium)	values taken from Fig. 2	[23]
Infectious bronchitis coronavirus (IBV A-5968)	56	5	5	1.0000	liquid (diluted medium)	values taken from Fig. 2	[23]
Infectious bronchitis coronavirus (IBV Be-42)	56	5	4.76	0.9520	liquid (diluted medium)	values taken from Fig. 2	[23]
Infectious bronchitis coronavirus (IBV Connaught)	56	5	4.13	0.8260	liquid (diluted medium)	values taken from Fig. 2	[23]
Infectious bronchitis coronavirus (IBV Holte)	56	5	3.96	0.7920	liquid (diluted medium)	values taken from Fig. 2	[23]
Infectious bronchitis coronavirus (IBV IB-41)	56	5	3.96	0.7920	liquid (diluted medium)	values taken from Fig. 2	[23]
Infectious bronchitis coronavirus (IBV Iowa-609)	56	5	4.44	0.8880	liquid (diluted medium)	values taken from Fig. 2	[23]
Infectious bronchitis coronavirus (IBV Ishida)	56	5	2.99	0.5980	liquid (diluted medium)	values taken from Fig. 2	[23]
Infectious bronchitis coronavirus (IBV KH)	56	5	2.78	0.5560	liquid (diluted medium)	values taken from Fig. 2	[23]
Infectious bronchitis coronavirus (IBV Nerima)	56	5	3.09	0.6180	liquid (diluted medium)	values taken from Fig. 2	[23]

Virus	Temperature [°C]	Exposition duration [min]	Log-reduction	k [1/min]	Sample condition	Remark	Reference*
Infectious bronchitis coronavirus (IBV Shiga)	56	5	2.76	0.5520	liquid (diluted medium)	values taken from Fig. 2	[23]
Middle East respiratory syndrome coronavirus (MERS-CoV)	38	60	1.34	0.0223	aerosol at 24% relative humidity	values taken from Fig. 4	[23]
Middle East respiratory syndrome coronavirus (MERS-CoV FRA2)	56	25	4	0.1600	liquid (DMEM +5% FCS)	values taken from abstract	[38]
Middle East respiratory syndrome coronavirus (MERS-CoV FRA2)	65	1	4	4.0000	liquid (DMEM +5% FCS)	values taken from abstract	[38]
Murine coronavirus (MHV A59)	37	5,760	1.59	0.0003	liquid (EMEM +5% FCS)	value taken from difference between 1 and 5 days in Fig. 3	[18]
Murine coronavirus (MHV 2)	37	1,440	3.3	0.0023	liquid (MEM +5% FCS)	value taken from text (mean of both results)	[39]
Murine coronavirus (MHV A59/ts379)	39.5	180	1.89	0.0105	liquid (DMEM +8% FCS)	values taken from Tab. 1, mutant strain	[24]
Murine coronavirus (MHV A59/wt)	39.5	180	0.92	0.0051	liquid (DMEM +8% FCS)	values taken from Tab. 1	[24]
Murine coronavirus (MHV 2)	40	30	0.26	0.0087	liquid (MEM +2% FCS)	values taken from Tab. 3	[44]
Murine coronavirus (MHV N)	40	30	0.28	0.0093	liquid (MEM +2% FCS)	values taken from Tab. 3	[44]
Murine coronavirus (MHV)	40	7,200	5	0.0165	surface and 20% RH	k values taken from Tab. 3 (and converted to minutes)	[29]
Murine coronavirus (MHV)	40	1,440	4	0.1404	surface and 50% RH	k values taken from Tab. 3 (and converted to minutes)	[29]
Murine coronavirus (MHV)	40	360	3	0.2658	surface and 80% RH	k values taken from Tab. 3 (and converted to minutes)	[29]
Murine coronavirus (MHV 2)	56	5	3.5	0.7000	liquid (EMEM)	values taken from Tab. 1	[39]
Murine coronavirus (MHV 2)	56	30	4	0.1333	liquid (MEM +5% FCS)	value taken from text	[39]
Murine coronavirus (MHV A59)	56	15	2.63	0.1753	liquid (EMEM +5% FCS)	value taken from difference between 5 and 20 min in Fig. 2	[18]

Attachment to: Hessling M, Hoenes K, Lingenfelder C. Selection of parameters for thermal coronavirus inactivation – a data-based recommendation. GMS Hyg Infect Control. 2020;15:Doc16. DOI: 10.3205/dgkh000351
*References refer to the article

Virus	Temperature [°C]	Exposition duration [min]	Log-reduction	k [1/min]	Sample condition	Remark	Reference*
Murine coronavirus (MHV JHM)	56	10	unspecified		liquid suspension	not included in analysis	[40]
Murine coronavirus (MHV 2)	60	5	3.55	0.7100	liquid (MEM +2% FCS)	values taken from Tab. 3	[44]
Murine coronavirus (MHV N)	60	1	2.87	2.8700	liquid (MEM +2% FCS)	values taken from Tab. 3	[44]
Murine coronavirus (MHV)	65	15	not specified		liquid (human serum)	values taken from Tab. 1, not included in analysis	[41]
Porcine epidemic diarrhea virus (PEDV CV777)	40	119.9	1	0.0083	liquid (MEM)	decimal reduction time for pH 7.2 given by author	[28]
Porcine epidemic diarrhea virus (PEDV CV777)	40	15.9	1	0.0629	liquid (plasma with 8.5% protein)	decimal reduction time for pH 7.2 given by author	[28]
Porcine epidemic diarrhea virus (PEDV CV777)	44	71.2	1	0.0140	liquid (MEM)	decimal reduction time for pH 7.2 given by author	[28]
Porcine epidemic diarrhea virus (PEDV CV777)	44	8.6	1	0.1163	liquid (plasma with 8.5% protein)	decimal reduction time for pH 7.2 given by author	[28]
Porcine epidemic diarrhea virus (PEDV CV777)	44	10	0.3	0.0300	liquid (EMEM +10% FCS)	values taken from Tab. 3 for pH 7.5	[42]
Porcine epidemic diarrhea virus (PEDV CV777)	44	10	0.3	0.0300	liquid (porcine plasma)	values taken from Tab. 3 for pH 7.5	[42]
Porcine epidemic diarrhea virus (PEDV CV777)	48	10	1	0.1000	liquid (EMEM +10% FCS)	values taken from Tab. 3 for pH 7.5	[42]
Porcine epidemic diarrhea virus (PEDV CV777)	48	10	1.7	0.1700	liquid (porcine plasma)	values taken from Tab. 3 for pH 7.5	[42]
Porcine epidemic diarrhea virus (PEDV CV777)	48	24.9	1	0.0402	liquid (MEM)	decimal reduction time for pH 7.2 given by author	[28]
Porcine epidemic diarrhea virus (PEDV CV777)	48	2.8	1	0.3571	liquid (plasma with 8.5% protein)	decimal reduction time for pH 7.2 given by author	[28]
Porcine epidemic diarrhea virus (PEDV V215/78)	50	180	3.42	0.0190	liquid (EMEM)	value taken from Fig. 2	[17]
Porcine epidemic diarrhea virus (PEDV V215/78)	60	30	unspecified		liquid (EMEM)	not included in analysis	[17]
Porcine epidemic diarrhea virus (PEDV PC22A)	65.6	0.5	3.7	7.4000	liquid (maintenance medium)	values taken from Tab. 1	[43]

Attachment to: Hessling M, Hoenes K, Lingenfelder C. Selection of parameters for thermal coronavirus inactivation – a data-based recommendation. GMS Hyg Infect Control. 2020;15:Doc16. DOI: 10.3205/dgkh000351
*References refer to the article

Virus	Temperature [°C]	Exposition duration [min]	Log-reduction	k [1/min]	Sample condition	Remark	Reference*
Porcine epidemic diarrhea virus (PEDV CV777)	70		>4.2		liquid (EMEM +10% FCS)	spray drying, not included in analysis	[44]
Porcine epidemic diarrhea virus (PEDV PC22A)	76.7	0.08333	2.6	31.2012	liquid (maintenance medium)	values taken from Tab. 1	[43]
Porcine epidemic diarrhea virus (PEDV PC22A)	87.8	0.08333	4.3	51.6021	liquid (maintenance medium)	values taken from Tab. 1	[43]
Severe acute respiratory syndrome coronavirus (SARS-CoV HKU39849)	38	1,440	2.03	0.0014	surface and 80% RH	values taken from Fig. 3	[30]
Severe acute respiratory syndrome coronavirus (SARS-CoV HKU39849)	38	1,440	3.36	0.0023	surface and 95% RH	values taken from Fig. 3	[30]
Severe acute respiratory syndrome coronavirus (SARS-CoV Frankfurt 1)	56	30	>5.01		liquid (MEM)	values taken from Tab. 1, not included in analysis	[25]
Severe acute respiratory syndrome coronavirus (SARS-CoV Frankfurt 1)	56	30	1.93	0.0643	liquid (MEM +20% FCS)	values taken from Tab. 1	[25]
Severe acute respiratory syndrome coronavirus (SARS-CoV Hanoi)	56	5	5.8	1.1600	liquid (EMEM +10% FCS)	value taken from text	[45]
Severe acute respiratory syndrome coronavirus (SARS-CoV P9)	56	60	0.44	0.0073	liquid (EMEM +10% FCS)	values estimated from Tab. 2	[46]
Severe acute respiratory syndrome coronavirus (SARS-CoV Urbani)	56	10	3.96	0.3960	liquid (DMEM +10% FCS)	values taken from Fig. 1	[22]
Severe acute respiratory syndrome coronavirus (SARS-CoV Urbani)	56	10	4.05	0.4050	liquid (human serum)	values taken from Fig. 1	[26]
Severe acute respiratory syndrome coronavirus (SARS-CoV Utah)	58	10	1.16	0.1160	liquid (Medium +4% FCS)	mean values taken from Fig. 1	[47]
Severe acute respiratory syndrome coronavirus (SARS-CoV Frankfurt 1)	60	30	>5.01		liquid (MEM)	values taken from Tab. 1, not included in analysis	[25]

Attachment to: Hessling M, Hoenes K, Lingenfelder C. Selection of parameters for thermal coronavirus inactivation – a data-based recommendation. GMS Hyg Infect Control. 2020;15:Doc16. DOI: 10.3205/dgkh000351

*References refer to the article

Virus	Temperature [°C]	Exposition duration [min]	Log-reduction	k [1/min]	Sample condition	Remark	Reference*
Severe acute respiratory syndrome coronavirus (SARS-CoV Frankfurt 1)	60	30	>5.01		liquid (MEM +20% FCS)	values taken from Tab. 1, not included in analysis	[25]
Severe acute respiratory syndrome coronavirus (SARS-CoV Frankfurt 1)	60	30	>5		liquid (MEM +2% FCS)	values taken from Tab. 1, not included in analysis	[27]
Severe acute respiratory syndrome coronavirus (SARS-CoV Urbani)	60	10	3.39	0.3390	liquid (PBS)	values taken from Fig. 1B	[26]
Severe acute respiratory syndrome coronavirus (SARS-CoV Urbani)	60	10	3	0.3000	liquid (PBS +10% BSA)	values taken from Fig. 1B	[26]
Severe acute respiratory syndrome coronavirus (SARS-CoV Urbani)	65	2	3.67	1.8350	liquid (DMEM +10% FCS)	values taken from Fig. 2	[22]
Severe acute respiratory syndrome coronavirus (SARS-CoV Urbani)	65	10	>5.3		liquid (human serum)	values taken from Fig. 1A, not included in analysis	[26]
Severe acute respiratory syndrome coronavirus (SARS-CoV P9)	67	15	0.44	0.0293	liquid (EMEM +10% FCS)	values estimated from Tab. 2	[27]
Severe acute respiratory syndrome coronavirus (SARS-CoV Utah)	68	5	2.1	0.4200	liquid (Medium +4% FCS)	mean values taken from Fig. 1	[47]
Severe acute respiratory syndrome coronavirus (SARS-CoV P9)	75	15	0.9	0.0600	liquid (EMEM +10% FCS)	values estimated from Tab. 2	[46]
Severe acute respiratory syndrome coronavirus (SARS-CoV Urbani)	75	15	>4.5		liquid (DMEM +10% FCS)	values taken from Fig. 2, not included in analysis	[22]
Severe acute respiratory syndrome coronavirus 2 (SARS-CoV-2)	37	1,440	3.57	0.0025	liquid (virus transport medium)	values taken from appendix	[8]
Severe acute respiratory syndrome coronavirus 2 (SARS-CoV-2)	37	2,880	4.24	0.0015	liquid (human sputum)	values taken from Tab. 1	[9]

Attachment to: Hessling M, Hoenes K, Lingenfelder C. Selection of parameters for thermal coronavirus inactivation – a data-based recommendation. GMS Hyg Infect Control. 2020;15:Doc16. DOI: 10.3205/dgkh000351
*References refer to the article

Virus	Temperature [°C]	Exposition duration [min]	Log-reduction	k [1/min]	Sample condition	Remark	Reference*
Severe acute respiratory syndrome coronavirus 2 (SARS-CoV-2)	42	30	1.64	0.0547	liquid (human sputum)	values taken from Tab. 1	[9]
Severe acute respiratory syndrome coronavirus 2 (SARS-CoV-2)	56	10	2.96	0.2960	liquid (virus transport medium)	values taken from appendix	[8]
Severe acute respiratory syndrome coronavirus 2 (SARS-CoV-2 026V-03883)	56	30	5.59	0.1863	liquid (MEM +5% FCS)	values taken from Tab. 4	[10]
Severe acute respiratory syndrome coronavirus 2 (SARS-CoV-2 026V-03883)	56	30	>5		liquid (MEM +5% FCS +0,3% BSA)	values taken from Tab. 4, not included in analysis	[10]
Severe acute respiratory syndrome coronavirus 2 (SARS-CoV-2)	56	15	3.75	0.2500	liquid (human sputum)	values taken from Tab. 1	[9]
Severe acute respiratory syndrome coronavirus 2 (SARS-CoV-2)	56	15	3.37	0.2247	liquid (DMEM +5% FCS)	values taken from Tab. 1	[11]
Severe acute respiratory syndrome coronavirus 2 (SARS-CoV-2 USA_WA1/2020)	56	15	3.66	0.2440	liquid (DMEM +10% FCS)	values taken from Fig. 5 (15 min value seemed more reliable than 30 min value)	[12]
Severe acute respiratory syndrome coronavirus 2 (SARS-CoV-2)	56	10	3.63	0.3630	liquid (human serum)	values taken from Tab. 1	[11]
Severe acute respiratory syndrome coronavirus 2 (SARS-CoV-2 026V-03883)	60	60	>5		liquid (MEM +5% FCS)	values taken from Tab. 4, not included in analysis	[10]
Severe acute respiratory syndrome coronavirus 2 (SARS-CoV-2 026V-03883)	60	60	5.82	0.0970	liquid (MEM +5% FCS +0,3% BSA)	values taken from Tab. 4	[10]
Severe acute respiratory syndrome coronavirus 2 (SARS-CoV-2)	65	5	1.74	0.3480	liquid (nasopharyngeal samples)	values taken from Tab. 1	[11]
Severe acute respiratory syndrome coronavirus 2 (SARS-CoV-2)	65	15	>6		liquid (DMEM +5% FCS)	values taken from Tab. 1, not included in analysis	[11]

*References refer to the article

Virus	Temperature [°C]	Exposition duration [min]	Log-reduction	k [1/min]	Sample condition	Remark	Reference*
Severe acute respiratory syndrome coronavirus 2 (SARS-CoV-2)	70	1	1.46	1.4600	liquid (virus transport medium)	values taken from appendix	[8]
Severe acute respiratory syndrome coronavirus 2 (SARS-CoV-2)	80	60	>6		liquid (DMEM +10% FCS)	mean values taken from Fig. 1/ text, not included in analysis	[14]
Severe acute respiratory syndrome coronavirus 2 (SARS-CoV-2 026V-03883)	92	15	>6		liquid (MEM +5% FCS)	values taken from Tab. 4	[10]
Severe acute respiratory syndrome coronavirus 2 (SARS-CoV-2 026V-03883)	92	15	>6		liquid (MEM +5% FCS + 0,3% BSA)	values taken from Tab. 4, not included in analysis	[10]
Severe acute respiratory syndrome coronavirus 2 (SARS-CoV-2)	95	0.5	0.34	0.6800	liquid (nasopharyngeal samples)	values taken from Tab. 1, not included in analysis	[11]
Severe acute respiratory syndrome coronavirus 2 (SARS-CoV-2 USA_WA1/2020)	100	5	>5		liquid (DMEM +10% FCS)	values taken from Fig. 5	[12]
Severe acute respiratory syndrome coronavirus 2 (SARS-CoV-2)	121	15	>5		surface of face masks in saturated steam of an autoclave	values taken from Tab. 1 for 4 different face masks	[48]
Transmissible gastroenteritis virus (TGEV)	37	30	3	0.1000	liquid (medium +1% lamb serum)	value taken from Tab. 4	[49]
Transmissible gastroenteritis virus (TGEV D52)	39	80	3.2	0.0400	liquid (EMEM +2% FCS)	values taken from Tab. 1(a)	[19]
Transmissible gastroenteritis virus (TGEV)	40	7,200	3.5	0.0254	surface and 20% RH	k values taken from Tab. 3 (and converted to minutes)	[29]
Transmissible gastroenteritis virus (TGEV)	40	1,440	4.5	0.1880	surface and 50% RH	k values taken from Tab. 3 (and converted to minutes)	[29]
Transmissible gastroenteritis virus (TGEV)	40	360	3	0.1643	surface and 80% RH	k values taken from Tab. 3 (and converted to minutes)	[29]
Transmissible gastroenteritis virus (TGEV D52)	43	50	3.87	0.0774	liquid (EMEM +2% FCS)	values taken from Tab. 1(a)	[19]
Transmissible gastroenteritis virus (TGEV D52)	47	21	4.21	0.2005	liquid (EMEM +2% FCS)	values taken from Tab. 1(a)	[19]

Attachment to: Hessling M, Hoenes K, Lingenfelder C. Selection of parameters for thermal coronavirus inactivation – a data-based recommendation. GMS Hyg Infect Control. 2020;15:Doc16. DOI: 10.3205/dgkh000351
*References refer to the article

Virus	Temperature [°C]	Exposition duration [min]	Log-reduction	k [1/min]	Sample condition	Remark	Reference*
Transmissible gastroenteritis virus (TGEV)	50	30	3	0.1000	liquid (medium +1% lamb serum)	value taken from text	[49]
Transmissible gastroenteritis virus (TGEV D52)	51	5	4.38	0.8760	liquid (EMEM +2% FCS)	values taken from Tab. 1(a)	[19]
Transmissible gastroenteritis virus (TGEV)	56	10	7.5	0.7500	liquid (medium +1% lamb serum)	value taken from Tab. 4	[49]
Transmissible gastroenteritis virus (TGEV D52)	57	2	4.53	2.2650	liquid (EMEM +2% FCS)	values taken from Tab. 1(a)	[19]
